# Co-occurrence and clustering of health conditions at age 11: cross-sectional findings from the Millennium Cohort Study

**DOI:** 10.1136/bmjopen-2016-012919

**Published:** 2016-11-22

**Authors:** Kathryn R Hesketh, James Fagg, Graciela Muniz-Terrera, Helen Bedford, Catherine Law, Steven Hope

**Affiliations:** 1 Population, Policy and Practice, UCL Institute of Child Health, London, UK; 2 Wandsworth Borough Council, London, UK; 3 Centre for Dementia Prevention, University of Edinburgh, Edinburgh, UK

**Keywords:** Millennium Cohort Study, Co-occurrence, Clustering, Childhood

## Abstract

**Objectives:**

To identify patterns of co-occurrence and clustering of 6 common adverse health conditions in 11-year-old children and explore differences by sociodemographic factors.

**Design:**

Nationally representative prospective cohort study.

**Setting:**

Children born in the UK between 2000 and 2002.

**Participants:**

11 399 11-year-old singleton children for whom data on all 6 health conditions and sociodemographic information were available (complete cases).

**Main outcome measures:**

Prevalence, co-occurrence and clustering of 6 common health conditions: wheeze; eczema; long-standing illness (excluding wheeze and eczema); injury; socioemotional difficulties (measured using Strengths and Difficulties Questionnaire) and unfavourable weight (thin/overweight/obese vs normal).

**Results:**

42.4% of children had 2 or more adverse health conditions (co-occurrence). Co-occurrence was more common in boys and children from lower income households. Latent class analysis identified 6 classes: ‘normative’ (57.4%): ‘atopic burdened’ (14.0%); ‘socioemotional burdened’ (11.0%); ‘unfavourable weight/injury’ (7.7%); ‘eczema/injury’ (6.0%) and ‘eczema/unfavourable weight’ (3.9%). As with co-occurrence, class membership differed by sociodemographic factors: boys, children of mothers with lower educational attainment and children from lower income households were more likely to be in the ‘socioemotional burdened’ class. Children of mothers with higher educational attainment were more likely to be in the ‘normative’ and ‘eczema/unfavourable weight’ classes.

**Conclusions:**

Co-occurrence of adverse health conditions at age 11 is common and is associated with adverse socioeconomic circumstances. Holistic, child focused care, particularly in boys and those in lower income groups, may help to prevent and reduce co-occurrence in later childhood and adolescence.

**Strengths and limitations of this study**

- Although evidence about prevalence and predictors of individual adverse health conditions in children exist, this is one of the first papers to explore multiple health conditions in UK children and determine how this is influenced by a child's sociodemographic circumstances.
- We used a large population-based cohort study, the Millennium Cohort Study, to assess co-occurrence and clustering of six common health conditions in childhood (asthma, eczema, long-standing illness, injury, socioemotional difficulties and unfavourable weight).
- We were constrained by the available data and were only able to assess conditions common within the sample (prevalence >10%), though less common conditions were subsumed within the long-standing illness category.
- Analyses were based on complete cases, but we used sampling weights to account for sampling design and attrition, and very similar results from analyses using multiply imputed data indicate the robustness of our reported findings.

## Introduction

Promoting children's health is a public health priority.^1^ Poorer health in childhood is likely be detrimental to a child's social and academic development^2^ ^3^ and predicts later adverse health in adulthood.^4–6^ To date, studies have tended to explore health conditions in isolation, or the relationship between several health conditions, with studies reporting evidence of obesity and emotional well-being,^7^ and allergy-related conditions^8^ ^9^ frequently occurring together in children. Yet in contrast to awareness of comorbidities in adults,^10–12^ and clustering of health behaviours in adults and children,^13^ ^14^ relatively little is known about how health conditions occur together in children.^11^ Determining how health conditions ‘co-occur’, that is, how many health conditions a child experiences, and ‘cluster’, that is, how health conditions are associated,^15^ could be important for a child's future health and development^1^ and for health service provision.^16^

Children may suffer from a range of physical and mental health conditions. Non-communicable conditions such as atopy, obesity, long-standing illness and socioemotional difficulties are common in the UK and developed countries worldwide.^17^ Injury is also an important cause of morbidity and mortality during childhood.^18^ Broadly, the prevalence of many individual common adverse child health conditions tends to increase with increasing socioeconomic disadvantage,^19^ with children living in lower socioeconomic circumstances tending to have poorer health outcomes.^20^ Asthma, injury and socioemotional difficulties have been shown to be more common in children from less affluent backgrounds.^21^ ^22^ Being exposed to poorer home situations, including perceived stressful circumstances, and living in disadvantaged environments, has been linked to higher risk of injury^23^ and respiratory diseases such as asthma.^24^ ^25^ Likewise, the prevalence of obesity for children living in deprived areas in the UK is twice that of children who live in the least deprived areas.^26^ In contrast, eczema, one of the most common childhood health conditions,^27^ often shows a reverse social gradient, with higher prevalence in children from higher socioeconomic groups.^28^ ^29^

However, there is scant research as to whether these gradients remain when individual health conditions are considered in combination. Co-occurrence of adverse health conditions is also likely to be socially patterned, and multiple adverse health conditions in childhood may potentially present an additional burden for disadvantaged families. Limited evidence suggests that boys^30^ and children from some ethnic minorities^31^ may be at greater risk of having multiple adverse health conditions, independent of socioeconomic circumstances.

This study therefore sought to investigate the prevalence, co-occurrence and clustering of six common physical and mental health conditions (wheeze; eczema; injury; long-standing illness; unfavourable weight and socioemotional difficulties) in UK 11-year-old children. We hypothesised that co-occurrence would be more common in children living in poorer socioeconomic circumstances, and in boys.

## Methods

### Participants and design

Data were from the Millennium Cohort Study (MCS), a longitudinal study of children born in the UK between September 2000 and January 2002.^32^ Briefly, first contact with the cohort child and their caregivers was at age 9 months, with survey interviews carried out in the home by trained interviewers. Information was collected on 72% of those approached, resulting in an initial sample of 18 296 singleton infants. Data were subsequently collected when the children were aged 3, 5, 7 and 11 years old.^32^ At age 11, 13 039 singleton children were interviewed; our analyses used complete case data on six health conditions and sociodemographic characteristics from 11 399 of these children (87% of those participating at age 11). Ethical approval for MCS5 was granted by the Yorkshire and Humber Research Ethics Committee in July 2011 (reference number 11/YH/0203).

### Measures

#### Health conditions

The Children and Young People's Health Outcomes Framework (CYPHOF)^16^ ^33^ sets out a range of health and social conditions relevant to young people from birth to 18 years and is used as a resource for health and social care professionals to monitor children's physical and mental health outcomes. Using the data available in MCS, and the CYPHOF^16^ ^33^ and information about prevalence of health conditions in UK children^17^ ^18^ as a guide, we included six common physical and mental health conditions in analyses. Common here was defined as ≥10% prevalence in the sample (table 1). Four physical health conditions were parent reported: wheeze; eczema (lifetime prevalence); injury (where healthcare services were sought) and long-standing illness (excluding those measured in other conditions). Using objective measures of height and weight, we also derived an unfavourable weight condition (including thin, overweight and obesity) derived using children's z-body mass index (BMI) score and the International Obesity Task Force (IOTF) cut-points.^35^ Mental health was measured with the Strengths and Difficulties Questionnaire (SDQ).^36^ ^37^ We derived a total SDQ score using the emotional, peer, conduct disorder and hyperactivity subscales, dichotomising this total using the predefined cut-off for borderline/abnormal socioemotional behaviour to define socioemotional difficulties.^34^

**Table 1:** Measures of adverse health conditions and their derivation (N=11 399)

Condition	Method of measurement	Variable label (coding)
Wheeze	Main respondent report whether child had wheezing or whistling in the chest in the last 12 months.	Yes (1) or no (0)
Eczema	Main respondent report whether child had ever had eczema.	Yes (1) or no (0)
Long-standing illness	Main respondent report that child has a long-standing illness (eg, epilepsy, hearing or sight conditions, cancer). Conditions measured in separate indicators (wheeze, eczema, injury, overweight/obesity and conduct/emotional/social disorders) were excluded.	Yes (1) or no (0)
Injury	Main respondent report of an unintentional injury for which medical attention was sought between MCS Sweep 4 (age 7) and Sweep 5 (age 11).	Yes (1) or no (0)
Unfavourable weight	Objective BMI (weight/height^2^) derived from heights and weights measured by trained interviewers; age and sex standardised BMI z-scores derived. BMI z-score classified according to the IOTF published cut-offs: thin^35^ (≤grade 1), overweight (excluding obesity) and obesity^35^ combined into unfavourable weight category.	*Index:* Unfavourable weight (thin, overweight or obese) (1) Normal (0) *LCA analysis:* Categories: thin, normal BMI, overweight, or obese
Socioemotional difficulties	Main respondent completed the SDQ questionnaire about the child. Sum of ‘emotional’, ‘peer’, ‘conduct’ and ‘hyperactivity’ SDQ subscales dichotomised based on published recommendations.^34^	Borderline/abnormal (≥14; 1) Normal (0–13; 0)

BMI, body mass index; SDQ, Strengths and Difficulties Questionnaire.

#### Sociodemographic characteristics

We chose four sociodemographic variables to explore concurrent associations with co-occurrence. Stable measures, that is, child's sex and ethnic group, were recorded at 9 months. Highest maternal educational qualifications and quintiles of household income (calculated using a modified OECD equivalence scale) when the cohort child was age 11 were also used.

### Analysis

We explored co-occurrence and clustering of health conditions using two complementary methods, to determine if the probability of having multiple adverse conditions differed by sex, ethnicity and socioeconomic variables.

#### Co-occurrence of adverse health conditions

We first derived a co-occurrence index by summing the total number of adverse health conditions each child had. For the purposes of the index, we combined thin and overweight (including obesity) into an ‘unfavourable weight’ category. This was because, although the conditions have different aetiologies and consequences, the three weight status categories are mutually exclusive and cannot therefore be counted as independent conditions. We used multinomial logistic regression to examine differences between children with one adverse health condition (the modal category) compared to those with 0, 2, 3 or 4 or more adverse conditions. We conducted univariable analyses to explore the association between co-occurrence and sex, ethnic group, highest level of maternal education and quintile of household income. We then mutually adjusted for all four sociodemographic variables, again using children with one adverse health condition as the reference category. All analyses were conducted using STATA V.13/SE (StataCorp LP. STATA13/SE. 2013).

We tested for collinearity between sociodemographic variables by removing each of the four assess variables in turn from the multivariable models and assessing SEs. We also carried out a number of sensitivity analyses. First, we assessed a dichotomised weight status variable (‘overweight or obese’ children vs ‘thin or normal range BMI’ children) in the co-occurrence index, as compared to the unfavourable weight category (thin, overweight and obese vs normal). Second, we explored the influence of including and excluding eczema in the index, as this was a lifetime prevalence rather than current indicator of an adverse health condition. Finally, children participating in MCS at age 11 were more likely to be white and from higher income homes compared to children recruited into the MCS at 9 months; this may potentially introduce bias when estimating the prevalence of adverse health conditions and co-occurrence. Although our analyses were weighted to take this attrition up to age 11 into account (see below), we also used multiple imputation to examine whether using complete case analysis biased our findings. We imputed data for all 18 296 children who were originally included at MCS1 (9 months) to assess the influence of attrition and item missing, repeating the co-occurrence analyses under the assumption that data were missing at random (see online supplementary material).^38^

10.1136/bmjopen-2016-012919.supp1 supplementary data

#### Clustering of adverse health conditions

We used latent class analysis (LCA)^39^ to identify how the six commonly occurring adverse health conditions clustered together. LCA is a data-driven approach*,* with individuals assigned to a class based on estimated probability of membership.^40^ The six health conditions and covariates (sex, ethnicity, highest maternal qualification and household income quintiles) were entered into the LCA model simultaneously, following recommended procedures.^39^ ^41^ LCA estimates two parameters: (1) the probability of children in the sample being allocated to a class and (2) the probability of children within a class experiencing each of the adverse health conditions (the item-response probabilities), adjusted for covariates.^42^ We used the co-occurrence index to constrain children with 0 or 1 adverse health condition to one class, allowing us to compare children with and without co-occurrence in the LCA.

As the ideal number of classes is not known a priori, we fitted a series of models with an increasing number of classes (from four to six).^39^ We then chose the best model, that is, the optimum number of classes, based on fit (Bayesian Information Criterion (BIC)^43^), classification (entropy)^44^ and by considering the interpretability of the classes. BIC takes account of fit and degrees of freedom, and models with a BIC closest to zero are considered the most statistically parsimonious.^43^ Entropy captures how well individuals are classified into each latent class. Ranging from 0 to 1, entropy of 0.8 or greater indicates good classification.^44^

We assigned labels to classes based on homogeneity and class separation.^42^ A latent class is homogenous when children within a given class have higher probabilities of experiencing the same adverse health conditions.^42^ Class separation is characterised by children within a class having a higher or lower probability of experiencing an adverse condition, relative to children in other classes.^42^ In order to check our classes were stable, we also conducted a sensitivity analysis, rerunning the LCA using two randomly split halves of the data set.

Response weights, the derivation of which have been described in detail elsewhere,^45^ were used in all analyses. These take into account the sampling design used in MCS and also attrition up to MCS5 (age 11). LCA was conducted using LATENT GOLD 5.0.

## Results

### Co-occurrence of adverse health conditions

The prevalence of the six adverse health conditions ranged from 12.0% for wheeze to 38.7% for injury (table 2). A total of 78% of children had one or more adverse health conditions; the modal category was one adverse condition (35.3%) and children with two or more co-occurring conditions comprised 42.6% of the total sample (table 3). Sixty-five different combinations of the six conditions were identified, with the three most common co-occurring conditions being unfavourable weight and injury, eczema and injury and eczema and unfavourable weight.

**Table 2:** Prevalence of co-occurrence by sociodemographic factors (n=11 399)

	Sample	Wheeze	Eczema	Long-standing illness	Injury	Socioemotional difficulties	Unfavourable weight
Overall prevalence % (n)		12.0 (1397)	30.9 (3585)	13.2 (1497)	38.7 (4328)	16.6 (1724)	33.7 (3680)
Sex*							
Male	51.6 (5766)	14.6 (862)	31.3 (1816)	15.6 (882)	41.1 (2352)	20.0 (1032)	31.4 (1856)
Female	48.4 (5633)	9.4 (262)	30.8 (1769)	10.8 (615)	36.3 (1976)	13.0 (692)	36.1 (2115)
Ethnicity**							
White	87.1 (9770)	12.0 (1191)	31.3 (3114)	13.5 (1323)	39.9 (3870)	16.6 (1460)	32.8 (3229)
Mixed	3.3 (308)	11.7 (36)	30.8 (95)	16.7 (49)	37.8 (113)	19.6 (51)	39.7 (119)
Indian	1.6 (234)	15.5 (40)	31.3 (81)	6.3 (15)	30.0 (78)	18.2 (37)	37.4 (107)
Pakistani	4.0 (643)	13.3 (96)	30.8 (222)	9.2 (64)	25.1 (181)	14.6 (117)	43.5 (312)
Black	3.0 (322)	12.1 (41)	31.3 (107)	10.9 (32)	27.6 (93)	13.0 (36)	42.3 (149)
Other	1.0 (122)	8.8 (12)	30.8 (43)	12.8 (14)	20.7 (29)	20.8 (23)	34.5 (55)
Maternal education*							
Degree/more	16.1 (2192)	12.5 (274)	35.3 (768)	12.0 (258)	36.4 (783)	6.7 (147)	25.6 (572)
Diploma	8.4 (1062)	11.3 (120)	33.4 (355)	11.8 (126)	36.1 (382)	10.6 (109)	31.2 (338)
A-levels	9.3 (1196)	11.5 (138)	29.8 (349)	12.4 (151)	36.9 (411)	11.7 (136)	30.3 (361)
GCSE	48.1 (5133)	12.0 (616)	31.2 (1589)	13.9 (699)	40.7 (2035)	18.2 (864)	35.6 (1886)
None	18.0 (1816)	12.6 (229)	26.8 (459)	13.7 (235)	38.2 (632)	26.7 (428)	38.3 (710)
Income quintiles*							
Q1 (high)	20.0 (2187)	11.7 (258)	34.2 (754)	13.0 (281)	37.4 (810)	7.9 (159)	28.4 (621)
Q2	19.9 (2355)	10.6 (254)	32.9 (802)	10.9 (266)	37.7 (881)	9.6 (216)	30.6 (737)
Q3	20.0 (2433)	12.0 (299)	31.4 (790)	13.2 (323)	40.0 (970)	12.9 (310)	35.2 (872)
Q4	20.5 (2275)	13.4 (314)	29.6 (668)	14.3 (322)	39.5 (884)	22.6 (465)	36.8 (866)
Q5 (low)	19.7 (2149)	12.5 (282)	27.8 (571)	14.7 (305)	38.7 (783)	28.9 (574)	36.6 (875)

χ^2^ test: *p<0.001, **p<0.05.

**Table 3:** Associations between co-occurrence index and sociodemographic circumstances at age 11 (n=11 399)

	Unadjusted relative risk ratio (95% CI)					Mutually adjusted relative risk ratio (95% CI)				
	0	1	2	3	4+	0	1	2	3	4+
Prevalence (%)	**22.1**	**35.2**	**25.2**	**11.8**	**5.7**	**22.1**	**35.2**	**25.2**	**11.8**	**5.7**
Sex (ref: boys)										
Girls	1.06 (0.96 to 1.18)	1.00	**0.88 (0.79 to 0.97)**	**0.78 (0.68 to 0.90)**	**0.56 (0.45 to 0.70)**	1.06 (0.96 to 1.18)	1.00	**0.88 (0.79 to 0.97)**	**0.78 (0.68 to 0.90)**	**0.56 (0.45 to 0.70)**
Ethnicity (ref: white)										
Mixed	0.87 (0.60 to 1.27)	1.00	1.03 (0.94 to 1.82)	0.92 (0.58 to 1.47)	1.72 (1.06 to 2.82)	0.89 (0.61 to 1.29)	1.00	1.28 (0.92 to 1.78)	0.89 (0.56 to 1.42)	1.61 (0.99 to 2.63)
Indian	0.93 (0.60 to 1.46)	1.00	0.92 (0.65 to 1.31)	0.94 (0.41 to 2.18)	0.57 (0.24 to 1.35)	0.91 (0.58 to 1.43)	1.00	0.91 (0.64 to 1.30)	0.92 (0.40 to 2.10)	0.54 (0.23 to 1.30)
Pakistani	0.93 (0.60 to 1.17)	1.00	0.81 (0.65 to 1.02)	**0.55 (0.38 to 0.80)**	**0.55 (0.34 to 0.90)**	1.02 (0.80 to 1.30)	1.00	**0.73 (0.57 to 0.94)**	**0.45 (0.30 to 0.68)**	**0.40 (0.25 to 0.67)**
Black	0.92 (0.71 to 1.19)	1.00	0.93 (0.68 to 1.27)	0.76 (0.41 to 1.42)	0.83 (0.50 to 1.39)	0.94 (0.72 to 1.22)	1.00	0.87 (0.64 to 1.20)	0.70 (0.37 to 1.30)	0.71 (0.41 to 1.20)
Other	0.81 (0.52 to 1.27)	1.00	0.87 (0.47 to 1.61)	0.59 (0.27 to 1.27)	**0.22 (0.09 to 0.54)**	0.82 (0.52 to 1.27)	1.00	0.85 (0.46 to 1.56)	0.54 (0.25 to 1.19)	**0.20 (0.09 to 0.46)**
Mat. Ed. (ref: higher/degree)										
Diploma	0.98 (0.82 to 1.17)	1.00	1.22 (1.04 to 1.57)	0.85 (0.67 to 1.08)	1.40 (0.96 to 2.17)	0.98 (0.82 to 1.17)	1.00	**1.16 (1.02 to 1.53)**	0.81 (0.63 to 1.03)	1.26 (0.90 to 1.76)
A-levels	1.03 (0.86 to 1.25)	1.00	1.13 (0.87 to 1.30)	1.02 (0.77 to 1.33)	1.42 (0.98 to 2.11)	1.02 (0.84 to 1.23)	1.00	1.05 (0.85 to 1.29)	0.95 (0.70 to 1.27)	1.30 (0.86 to 1.92)
GCSEs	0.85 (0.75 to 1.00)	1.00	**1.24 (1.07 to 1.42)**	**1.41 (1.19 to 1.68)**	**1.64 (1.20 to 1.68)**	0.88 (0.75 to 1.02)	1.00	1.07 (0.92 to 1.26)	1.22 (0.99 to 1.52)	1.23 (0.88 to 1.70)
None	0.87 (0.71 to 1.06)	1.00	**1.22 (0.99 to 1.50)**	**1.41 (1.10 to 1.82)**	**1.98 (1.39 to 2.80)**	0.97 (0.78 to 1.22)	1.00	1.06 (0.83 to 1.34)	1.22 (0.90 to 1.63)	1.38 (0.98 to 2.08)
Income (ref: Q1 (high))										
Q2	1.12 (0.95 to 1.32)	1.00	1.08 (0.92 to 1.27)	1.09 (0.88 to 1.36)	1.02 (0.73 to 1.45)	1.16 (0.98 to 1.38)	1.00	1.05 (0.89 to 1.24)	1.01 (0.83 to 1.32)	0.92 (0.63 to 1.34)
Q3	1.08 (0.93 to 1.27)	1.00	**1.26 (1.06 to 1.50)**	**1.37 (1.13 to 1.67)**	**1.48 (1.10 to 2.00)**	1.16 (0.97 to 1.38)	1.00	1.21 (1.00 to 1.46)	**1.22 (1.02 to 1.63)**	1.36 (0.96 to 1.94)
Q4	0.88 (0.72 to 1.06)	1.00	**1.31 (1.09 to 1.57)**	**1.40 (1.12 to 1.77)**	**1.77 (1.29 to 2.42)**	0.93 (0.76 to 1.15)	1.00	1.23 (1.00 to 1.52)	1.21 (0.93 to 1.59)	**1.56 (1.07 to 2.28)**
Q5 (low)	0.86 (0.71 to 1.05)	1.00	**1.30 (1.07 to 1.57)**	**1.53 (1.24 to 1.89)**	**1.91 (1.40 to 2.61)**	0.90 (0.71 to 1.54)	1.00	1.23 (0.97 to 1.55)	**1.34 (1.01 to 1.77)**	**1.72 (1.14 to 2.60)**

Mutually adjusted model: adjusted for all other covariates; bold indicates significant result.

GCSE: General Certificate of Secondary Education.

Multinomial regression analyses were used to estimate a relative risk ratio for children with no or co-occurring health conditions compared to those with one adverse health condition. That is, a relative risk ratio of <1 indicates that risk is higher in children with one health condition, and when >1, the risk is higher in those with no or co-occurring health conditions. In unadjusted and adjusted models, children with 0 or 1 adverse health condition did not differ significantly by sex, ethnicity or socioeconomic variables. In unadjusted analyses, boys, children of mothers with lower educational attainment and those living in lower income households were more likely to have co-occurring conditions compared to children with one adverse health condition. The association remained for boys and children from lower income homes in mutually adjusted models, suggesting that even after adjusting for other sociodemographic variables, boys and children from lower income homes are more likely to have co-occurring conditions (table 3).

Our tests for collinearity showed only small increases in the SEs compared to the fully adjusted model, suggesting that the association between sociodemographic variables had little impact on our findings. The use of a dichotomous weight status variable (‘overweight or obese’ children vs ‘thin or normal range BMI’ children) in the co-occurrence index and, separately, removal of the eczema variable made only small differences to the percentages of children in each of the co-occurrence index categories; the patterns of results by sex, ethnicity or socioeconomic variables. The use of multiple imputation did not substantially change prevalences of health conditions or the distribution of the co-occurrence index from those using complete cases (see online supplementary material and tables S3–S5).

### Clustering of adverse health conditions

The LCA fit statistics indicated that a 6-class model was most appropriate, with five latent subgroups or ‘classes’ emerging from analyses, in addition to the one constrained class (children with 0 or 1 adverse health condition) (table 4). Classification was good (entropy of 0.9), and sensitivity analyses using a split sample showed that these classes were stable.

**Table 4:** Prevalence and subgroup specific probabilities of class membership by adverse health conditions and sociodemographic characteristics at age 11 (n=11 399)

	Normative	Atopic burdened	Socioemotional burdened	Unfavourable weight/injury	Eczema/ injury	Eczema/ unfavourable weight
Class size	0.57	0.14	0.11	0.07	0.07	0.04
Wheeze						
No	0.98	0.40	0.90	0.96	0.93	0.93
Yes	0.02	0.56	0.10	0.04	0.07	0.07
Eczema (ever)						
No	0.87	0.42	0.62	0.77	0.00	0.00
Yes	0.13	0.58	0.38	0.23	1.00	1.00
Injury						
No	0.78	0.51	0.49	0.00	0.00	0.99
Yes	0.22	0.49	0.51	1.00	1.00	0.01
Long-standing illness						
No	0.98	0.41	0.68	0.97	0.98	0.97
Yes	0.02	0.59	0.32	0.03	0.02	0.03
SDQ total						
Normal	0.96	0.80	0.00	0.99	0.98	0.97
Abnormal	0.04	0.20	1.00	0.01	0.02	0.03
BMI						
Normal	0.80	0.60	0.58	0.00	0.70	0.00
Thin	0.02	0.07	0.07	0.20	0.04	0.20
Overweight	0.13	0.26	0.27	0.63	0.19	0.66
Obese	0.06	0.06	0.07	0.17	0.07	0.14
Index						
0	0.39	0.00	0.00	0.00	0.00	0.00
1	0.61	0.00	0.00	0.00	0.00	0.00
2	0.00	0.46	0.49	0.82	0.65	0.86
3	0.00	0.30	0.32	0.17	0.33	0.13
4/+	0.00	0.24	0.19	0.01	0.02	0.01
*Covariates*						
Sex						
Girls	0.51	0.42	0.36	0.54	0.50	0.60
Boys	0.49	0.58	0.64	0.46	0.50	0.40
Ethnicity						
White	0.87	0.88	0.88	0.89	0.93	0.79
Mixed	0.03	0.03	0.04	0.03	0.03	0.07
Indian	0.02	0.01	0.02	0.01	0.00	0.04
Pakistani/Bangladeshi	0.05	0.04	0.03	0.03	0.01	0.05
Black	0.03	0.04	0.02	0.02	0.02	0.04
Other	0.01	0.00	0.01	0.01	0.01	0.01
Maternal qualifications						
Higher/degree	0.22	0.22	0.07	0.15	0.23	0.21
Diploma	0.09	0.09	0.05	0.08	0.10	0.10
A-levels	0.10	0.09	0.06	0.09	0.08	0.09
GCSEs	0.46	0.49	0.52	0.47	0.48	0.51
None	0.13	0.11	0.30	0.21	0.11	0.08
Income quintiles						
Q1 (high)	0.22	0.23	0.05	0.17	0.26	0.19
Q2	0.21	0.18	0.11	0.21	0.22	0.24
Q3	0.20	0.21	0.15	0.26	0.21	0.24
Q4	0.19	0.21	0.31	0.20	0.15	0.21
Q5 (low)	0.18	0.16	0.38	0.16	0.17	0.12

Classes are labelled to reflect the relationships between the predominant adverse health conditions within each class. Classes can be described as follows: normative: constrained to children with no or one condition only; atopic burdened: higher probability of wheeze, eczema, long-standing illness and of socioemotional difficulties and overweight/obesity; socioemotional burdened: higher probability of being at risk of socioemotional difficulties, and injury and overweight/obesity; Unfavourable weight/injury: higher probability of unhealthy weight and injury; eczema/injury: higher probability of eczema and injury; eczema/unfavourable weight: higher probability of eczema and unhealthy weight.

BMI, body mass index; SDQ, Strengths and Difficulties Questionnaire; GCSE: General Certificate of Secondary Education.

The largest proportion of children (57.0%) were allocated to the ‘normative’ constrained class, with the probability of these children having no and one adverse health condition being 0.39 and 0.61, respectively. Injury and eczema were common conditions in children with only one adverse condition whereas wheeze, long-standing illness and socioemotional difficulties were not. Children in the ‘atopic burdened’ class (14.0% of the sample) were more likely to have wheeze, eczema and long-standing illness, as well as socioemotional difficulties and unfavourable weight. These children were also more likely to be boys. Children with multiple adverse health conditions (3 or more) were more likely to be in the multiple-condition ‘atopic burdened’ and ‘socioemotional burdened’ classes. Those in the ‘socioemotional burdened’ class (11.0%) had a high probability of experiencing socioemotional difficulties, in addition to long-standing illness and overweight/obesity; they were more likely to be boys, have mothers with lower educational attainment and live in lower income households.

Three classes, ‘unfavourable weight/injury’ (8.0%), ‘eczema/injury’ (6.0%) and ‘eczema/unfavourable weight’ (4.0%), were more likely to be composed of children with two adverse health conditions. These classes mirrored the three most common combinations of two adverse health conditions in the co-occurrence index.

## Discussion

Over three-quarters of 11-year-old children in this sample (78%) had at least one adverse health condition, and 43% experienced co-occurrence (two or more conditions). Co-occurrence differed by sex and was socially patterned, with boys and children living in lower income households being at higher risk of having multiple adverse health conditions. LCA identified five distinct classes of conditions, in addition to the group of children with no or one adverse health condition: ‘atopic burdened’, ‘socioemotional burdened’, ‘unfavourable weight/injury’, ‘eczema/injury’ and ‘eczema/unfavourable weight’. Children identified by the index as having greater numbers of adverse health conditions were more likely to be in the ‘atopic burdened’ and ‘socioemotional burdened’ classes; these children were more likely to be boys, have mothers with lower educational attainment and live in lower income households. Children in ‘unfavourable weight/injury’, ‘eczema/injury’ (6.0%) and ‘eczema/unfavourable weight’ classes were more likely to have these two adverse health conditions alone, with girls more likely to be in the ‘eczema/unfavourable weight’ class. Children in the ‘unfavourable weight/injury’ class were also more likely to have mothers with lower educational attainment. Despite the use of different analytic techniques, similarities identified in co-occurrence and clustering of adverse health conditions suggest that occurrence of multiple adverse health conditions in children is patterned by gender and socioeconomic circumstances.

Previous studies have explored the relationship between one or two health conditions, reporting evidence for co-occurrence or clustering of obesity and emotional well-being,^7^ and allergy-related conditions.^8^ ^9^ Few have assessed co-occurrence and clustering of multiple adverse health conditions in children.^11^ ^30^ A study in Scotland found low levels of multimorbidity in a sample of 0–24-year-olds (1.9% of 479 156 participants),^11^ but explored 40 health conditions commonly reported in adults. In contrast, this study used six conditions identified as important to children and young people,^16^ ^33^ analysed in a population-based cohort. Given that we found that over 40% of children were likely to have co-occurrence, the prevalence of children experiencing multiple common adverse health conditions in the UK may be higher than previously estimated.^11^

We identified several classes (ie, ‘atopic burdened’ and ‘socioemotional burdened’) that support earlier work suggesting that atopic conditions,^8^ ^30^ and injuries and socioemotional difficulties,^46^ and obesity and emotional well-being^7^ occur together in childhood. However, while these previous studies have sought to identify the link between two or three conditions only, we used a data-driven approach to identify how multiple adverse health conditions clustered together; this resulted in more than two conditions being included in any one cluster. As in a nationally representative sample of US 4-year-old children which also used LCA to identify adverse health clusters,^30^ boys in our sample were more likely to have co-occurring conditions. They were also more likely to be classified into the ‘atopic burdened’ and ‘socioemotional burdened’ classes, in part driven by a higher prevalence of individual adverse conditions, with wheeze and risk of socioemotional difficulties both twice as likely to occur in boys compared to girls. As LCA is a data-driven approach, the clusters derived here reflect groupings of health conditions in the MCS sample. Although our findings are similar to those seen previously and may reflect meaningful clusters of health conditions in children, further research is required to determine the stability of these groupings across other samples and time.

Evidence from this research and the US study^30^ identified social patterning in co-occurrence and clustering of adverse health conditions, highlighting that inequalities are apparent in early life,^30^ may persist into later childhood. Here, there appeared to be a gradient in prevalence of co-occurrence by child's socioeconomic circumstances such that children living in lower income homes were at greater risk of having multiple co-occurring conditions. The association was similar using maternal education attainment and household income measured at age 11 (though not significant in the former). Given maternal educational attainment changed very little from the child's birth to age 11, this may be a more stable indicator of socioeconomic circumstances, whereas income may be more likely to vary over time. This may partially explain why maternal educational attainment and household income variables show only a moderate correlation in MCS (r=0.63). Nevertheless, for unadjusted and adjusted analyses, children in the lowest income quintiles appeared to be at increased risk of having multiple co-occurring conditions. Further research is now required to explore possible mechanisms through which socioeconomic circumstances during early and later childhood may lead to multiple adverse conditions.

### Strengths and limitations

To the best of our knowledge, this is one of the first studies to assess co-occurrence and clustering of commonly occurring adverse health conditions in UK children, using conditions identified as relevant for young people from the CYPHOF.^16^ ^33^ While cohort studies are longitudinal, the large sample size and national representativeness of the MCS make individual sweeps a useful source of cross-sectional data. Data were from 11 399 children, in contrast to other sources such as the Health Survey for England where data are available for fewer than 500 11-year-olds per sweep.^47^ However, we were constrained by the available data and were only able to assess common conditions. Other conditions such as epilepsy, cerebral palsy, cancer and deafness were grouped together within the long-standing illness category; this served to capture less common conditions that nevertheless contribute to disease burden and may impact children's quality of life, thus providing adequate statistical power for analysis. As with most longitudinal studies, attrition and missing data in MCS may have led to bias in our findings. Although our reported analyses were based on complete cases, sampling weights were used to account for sample design and attrition in MCS up to age 11. Moreover, results from analyses using multiply imputed data indicated the robustness of our reported co-occurrence findings, as the prevalence estimates and relative risk ratios were very similar for complete case and imputed analyses. This suggests that our co-occurrence findings are generalisable to the original MCS study population. While LCA is helpful in identifying common classes of children, it may mask the complexity of how health conditions cluster, with 65 different combinations of adverse conditions identified in these children. This work therefore illustrates the benefit of using different methods to assess multiple adverse health conditions.

Although previous studies have explored one or two health conditions, we were able to explore a wider range of adverse health conditions and consider physical and mental health conditions, given their mutual importance for children's well-being.^1^ Weight status was assessed using objective measures of height and weight; the remaining physical and mental health conditions were assessed by main respondent report, generally the child's mother. Although the consequences of injuries may be time limited, this condition was included in the index as it has been identified as important to children's health and is indicative of children's contact with healthcare services.^33^ Eczema was assessed using a lifetime prevalence question. It is possible that this resulted in overestimation of the prevalence of eczema at age 11 if a child's symptoms had been resolved by age 11, although sensitivity analyses indicate that exclusion of eczema made little difference to our co-occurrence prevalences (ie, 2% overall). As asthma phenotypes vary,^48^ we used the presence of wheeze in the preceding 12 months as a condition, which may be indicative of persistent wheeze and/or asthma.^49^

Parent report may have been subject to bias, leading to either inflation or under-reporting of children's health conditions, and subsequent prevalence of co-occurrence. Although linked healthcare records are not yet available in the MCS, parents are likely to be very knowledgeable about their children's health conditions. Indeed, maternal report of long-standing illness has been shown to have a medium-to-high level of agreement with medical records.^50^ ^51^ Only one prior study has been conducted in the UK to explore ‘multimorbidity’ in children reporting a prevalence of <5%. This included a range of conditions more relevant to adults (ie, CHD, stroke). We would expect the prevalence to be higher using conditions relevant to children, which is what this study identified.

### Implications for policy and practice

Co-occurrence of two or more adverse health conditions is common in UK children, and the origins of co-occurrence appear to be socially patterned. As children in lower income households appear to face an additional burden of ill health compared to those in more affluent circumstances, they may require extra support from health services. That co-occurrence and clustering of adverse health conditions is prevalent in this age group emphasises the need for child and family centred care to be embedded in healthcare provision, including specialist and school services. Taking account of all aspects of a child's life may be particularly useful for boys, with gender-sensitive care providing an opportunity to narrow the apparent gender-gap in co-occurrence. Finally, policy and practice initiatives tend to focus on tackling single adverse health conditions in isolation; consideration of how adverse health conditions co-occur may result in improved chances of success.
