# Supplementary material for: Co-occurrence and clustering of health conditions at age 11: cross-sectional findings from the Millennium Cohort Study
Source: BMJ Open. 2016 Nov 22;6(11):e012919. doi: 10.1136/bmjopen-2016-012919 (PMC5128951; doi:10.1136/bmjopen-2016-012919)
Supplement: supplementary data [file bmjopen-2016-012919supp.pdf]

Supplementary Material

Cohort attrition

A total of 18,296 singleton infants provided valid data at MCS1 (9 months). The missingness associated with cohort attrition/ non-participation up to MCS5 is shown in Table S1. This compares those 18,296 children who provided data at MCS1 to those 13,112 11-year-old children who were interviewed at MCS5 (age 11).

Table S1 Cohort attrition up to MCS5 (Age 11)

	MCS1 Sample[a] % Composition	MCS5 Sample[a] % Composition
**Socio-demographic factors**		
**Sex**		
Male	51.5	50.4
Female	48.5	49.5
**Ethnicity**		
White	82.5	83.4
Mixed	3.0	2.8
Indian	2.5	2.5
Pakistani	6.9	7.0
Black	3.6	3.1
Other	1.5	1.3
**Maternal Educational Attainment**		
Degree/more	16.2	18.6
Diploma	8.6	9.1
A-levels	9.6	10.2
GCSE	45.5	44.5
None	20.1	17.6
**Household income quintiles**		
Q1 (high)	16.0	18.0
Q2	17.4	19.3
Q3	18.9	19.2
Q4	22.5	21.3
Q5 (low)	25.2	22.2

a: unweighted sample % prevalence

Item missingness

At MCS5 (age 11) a total of 13,112 parent-child pairs were interviewed; specific item missingness for those providing data at age 11 are shown in Table S2. As outlined in the paper, 11,399 children were subsequently included in the complete case analyses, providing data for all variables in Table S2.

Table S2 Item missingness at MCS5 (n=13,112)

	Missingness n (%)	Data provided n (%)
**Socio-demographic factors**		
Sex	71 (0.5)	13,085 (96.4)
Ethnicity	2 (0)	13,110 (100.0)
Maternal Educational Attainment	410 (3.1)	12,702 (96.9)
Household Income	506 (3.9)	12,606 (96.1)
**Adverse Health Conditions**[a]		
Wheeze	1 (0)	13,111 (100.0)
Eczema	112 (0.9)	13,000 (99.1)
Longstanding illness	100 (0.8)	13,002 (99.2)
Injury	115 (0.9)	12,997 (99.1)
Unfavourable weight	528 (4.0)	12,584 (96.0)
Total SDQ score	458 (3.5)	12,654 (96.5)

a: n=data were provided on 6 adverse health conditions for 11,399 children , n=1567 for 5 conditions, n=41 for 4 conditions 4, n=22 for 3 conditions, n=8 for 2 conditions and n=1 for 1 adverse health conditions.

Multiple imputation

We conducted multiple imputation using Multiple Imputation by Chained Equations (MICE) (mi command in STATA), assuming data were missing at random.[1] Data were imputed for all children participating in MCS1 (n=18,296) and we imputed for both cohort attrition (in up to n=5,712 children per variable) and item missingness (in up to n=528 children per variable).

The following variables were included in imputation analysis: wheeze; eczema; longstanding illness; injury; BMI; total SDQ score; sex; ethnicity; maternal education and income. We used a burn in of 20 and imputed 10 datasets for analyses.

Comparison of prevalence of adverse health conditions in complete case and imputed data sets are shown in Table S3, with prevalence of co-occurrence shown in Table S4. Multinomial regression analyses using the imputed datasets are shown in Table S5.

Table S3 Prevalence of adverse health outcomes comparing complete and imputed data

	**Weighted %<br>Complete (n=11,399)**	**Weighted %<br>Imputed (n=18,296)**
Wheeze	12.0	11.9
Eczema	30.9	30.0
Longstanding illness	13.2	13.0
Injury	38.7	36.8
BMI		
Thin	6.5	7.0
Normal BMI	66.3	65.3
Overweight	20.7	21.0
Obese	6.5	6.7
Total SDQ score	16.6	15.9

Table S4 Co-occurrence index at age 11 comparing complete and imputed data

	**Proportion of children**	
**Number of adverse outcomes**	**Complete (n=11,399)**	**Imputed (n=18,296)**
0	**22.1**	**22.6**
1	**35.2**	**36.0**
2	**25.2**	**24.7**
3	**11.8**	**11.1**
4/+	**5.7**	**5.5**

[1] Allison PD. Missing Data. *Quant Appl Soc Sci* 2001;104. doi:10.1136/bmj.38977.682025.2C

Table S5 Associations between co-occurrence index and socio-demographic circumstances at age 11 (imputed n=18,296)

	Unadjusted relative risk ratio (95% CI)					Mutually adjusted relative risk ratio (95% CI)				
	**0**	**1**	**2**	**3**	**4+**	**0**	**1**	**2**	**3**	**4+**
**Prevalence (%)**	**22.6**	**36.0**	**24.7**	**11.1**	**5.5**	**22.6**	**36.0**	**24.7**	**11.1**	**5.5**
**Sex (ref: Boys)**										
Girls	1.07 (0.96, 1.20)	1.00	**0.88 (0.79, 0.99)**	**0.79 (0.68, 0.91)**	**0.65 (0.52, 0.83)**	1.07 (0.95, 1.20)	1.00	**0.89 (0.80, 0.99)**	**0.80 (0.69, 0.93)**	**0.64 (0.51, 0.81)**
**Ethnicity (ref: White)**										
Mixed	0.89 (0.62, 1.30)	1.00	1.25 (0.90, 1.74)	0.97 (0.63, 1.50)	1.75 (1.10, 2.78)	0.91 (0.63, 1.29)	1.00	1.25 (0.89, 1.77)	0.89 (0.57, 1.40)	**1.64 (1.01, 2.66)**
Indian	0.93 (0.73, 1.50)	1.00	0.84 (0.61, 1.15)	0.81 (0.40, 1.67)	**0.42 (0.18, 0.92)**	0.94 (0.63, 1.40)	1.00	0.78 (0.55, 1.30)	0.92 (0.34, 1.72)	**0.40 (0.18, 0.90)**
Pakistani	0.93 (0.75, 1.16)	1.00	**0.71 (0.57, 0.90)**	**0.47 (0.31, 0.71)**	**0.50 (0.32, 0.78)**	1.00 (0.77, 1.29)	1.00	**0.66 (0.51, 0.85)**	**0.45 (0.27, 0.66)**	**0.33 (0.21, 0.52)**
Black	0.92 (0.70, 1.28)	1.00	0.89 (0.66, 1.21)	0.74 (0.44, 1.26)	0.75 (0.46, 1.20)	0.98 (0.74, 1.34)	1.00	0.86 (0.64, 1.17)	0.70 (0.36, 1.15)	**0.58 (0.34, 0.98)**
Other	0.81 (0.54, 1.55)	1.00	0.67 (0.38, 1.17)	0.55 (0.25, 1.19)	**0.22 (0.09, 0.54)**	0.97 (0.56, 1.69)	1.00	0.68 (0.37, 1.25)	0.54 (0.23, 1.16)	**0.17 (0.07, 0.43)**
**Mat. Ed. (ref: Higher/degree)**										
Diploma	1.00 (0.80, 1.25)	1.00	1.17 (0.93, 1.47)	1.00 (0.76, 1.33)	**1.62 (1.06, 2.46)**	0.99 (0.79, 1.25)	1.00	**1.11 (.088, 1.39)**	0.93 (0.69, 1.23)	1.41 (0.91, 2.18)
A-levels	1.02 (0.84, 1.25)	1.00	1.08 (0.87, 1.34)	1.01 (0.75, 1.36)	**1.62 (1.09, 2.41)**	1.01 (0.81, 1.23)	1.00	0.94 (0.75, 1.19)	0.91 (0.66, 1.27)	1.38 (0.89, 2.14)
GCSEs	0.90 (0.78, 1.05)	1.00	**1.26 (1.07, 1.48)**	**1.34 (1.12, 1.61)**	**1.84 (1.40, 2.44)**	0.93 (0.78, 1.12)	1.00	1.06 (0.88, 1.27)	1.10 (0.87, 1.39)	1.24 (0.88, 1.78)
None	0.92 (0.77, 1.11)	1.00	**1.27 (1.03, 1.56)**	**1.31 (1.04, 1.65)**	**2.37 (1.66, 3.38)**	1.04 (0.84, 1.29)	1.00	1.08 (0.84, 1.40)	1.10 (0.84, 1.45)	1.52 (0.95, 2.44)
**Income (ref: Q1 [High])**										
Q2	1.08 (0.91, 1.29)	1.00	1.13 (0.96, 1.33)	1.08 (0.87, 1.35)	1.05 (0.75, 1.46)	1.10 (0.92, 1.34)	1.00	1.15 (0.96, 1.36)	1.05 (0.83, 1.33)	1.01 (0.69, 1.46)
Q3	1.06 (0.89, 1.26)	1.00	**1.29 (1.08, 1.54)**	**1.27 (1.03, 1.56)**	**1.61 (1.19, 2.18)**	1.11 (0.92, 1.35)	1.00	1.32 (1.07, 1.61)	1.28 (0.99, 1.73)	**1.59 (1.10, 2.30)**
Q4	0.89 (0.74, 1.08)	1.00	**1.31 (1.09, 1.56)**	**1.37 (1.09, 1.71)**	**1.99 (1.44, 2.74)**	0.91 (0.73, 1.15)	1.00	1.37 (1.09, 1.70)	**1.35 (1.02, 1.78)**	**1.89 (1.24, 2.88)**
Q5 (low)	0.90 (0.74, 1.10)	1.00	**1.26 (1.05, 1.51)**	**1.32 (1.08, 1.62)**	**1.95 (1.41, 2.69)**	0.90 (0.70, 1.69)	1.00	1.36 (1.07, 1.72)	**1.43 (1.09, 1.87)**	**2.02 (1.28, 3.17)**

a: Mutually adjusted analysis adjusted for all other covariates in the table; GCSE: General Certificate of Secondary Education; Bold indicates significant result
